# When Hours Matter: A 24/7 Laboratory and Fast-Track Diagnostic Pathway for Blood Cultures in Critical Patients

**DOI:** 10.3390/antibiotics15050425

**Published:** 2026-04-23

**Authors:** Marta Corbella, Greta Petazzoni, Elena Seminari, Cristina Merla, Debora De Vitis, Elizabeth Iskandar, Alba Muzzi, Marco Rettani, Raffaele Bruno, Fausto Baldanti, Patrizia Cambieri, the San Matteo Pavia Microbiology and Virology Working Group

**Affiliations:** 1 Microbiology and Virology Unit, Fondazione IRCCS Policlinico San Matteo, 27100 Pavia, Italy; 2 Department of Clinical, Surgical, Diagnostic and Paediatric Sciences, University of Pavia, 27100 Pavia, Italy; 3 Infectious Diseases Unit, Fondazione IRCCS Policlinico San Matteo, 27100 Pavia, Italy; 4 Medical Direction, Fondazione IRCCS Policlinico San Matteo, 27100 Pavia, Italy; 5 Information Technology, Fondazione IRCCS Policlinico San Matteo, 27100 Pavia, Italy

**Keywords:** blood culture, sepsis, 24/7 laboratory organization, critical patients

## Abstract

**Background/Objectives**: Bloodstream infections are among the most severe infectious diseases, with mortality rates up to 25%. Delays as short as one hour in the diagnosis or initiation of the appropriate antimicrobial therapy can significantly worsen patient outcomes. **Methods**: This retrospective study, in an Italian 900-bed hospital from January 2019 to December 2024, evaluates the impact of a 24/7 reorganization of the clinical microbiology laboratory, adding a night shift to ensure around-the-clock processing and introducing a fast-track diagnostic pathway to prioritize the blood cultures from critically ill patients (called urgent blood cultures) in terms of turnaround times for Gram staining, microorganism identification, and resistance marker detection. **Results**: A total of 194,171 blood cultures were processed. Following the implementation of the 24/7 model, the median Gram stain turnaround time decreased from 4.46 to 1.40 h, microorganism identification turnaround time decreased from 5.75 to 2.35 h, and resistance marker turnaround time from 6.97 to 2.68 h. Significant reductions were observed especially during night shifts. Urgent blood cultures yielded a higher positivity rate (16.22% vs. 13.04%) and included the isolation of time-critical bacteria that can cause meningitis, such as Streptococcus pneumoniae. **Conclusions**: The continuous around-the-clock processing of blood culture and prioritized blood cultures for critically ill patients significantly reduced reporting times, particularly overnight. This model enhances early sepsis management and exemplifies how tailored and precision microbiology, supported by strong interdisciplinary collaboration and effective communication, can enhance earlier targeted antimicrobial treatment.

## 1. Introduction

Bloodstream infections (BSIs) are among the most severe infectious diseases, with mortality rates ranging from 11% to 25% [1]. Even a one-hour delay in reporting results and initiating or adjusting antibiotic therapy can negatively affect patient survival [1,2]. In cases of sepsis, survival rates drop by 7.6% for every hour of delay in initiating appropriate antibiotic therapy [2]. Sepsis guidelines recommend starting antimicrobial treatment within one hour for septic shock and within three hours for suspected sepsis without shock [3]. The early identification of high-risk patients using early warning scores (MEWS, NEWS, NEWS-2, qSOFA) and continuous 24/7 blood culture processing are essential to prevent diagnostic and therapeutic delays and improve patient outcomes [4].

Empirical broad-spectrum antimicrobial therapy in sepsis must be balanced against the growing threat of antimicrobial resistance, highlighting the need for effective antimicrobial stewardship programs [5]. Blood cultures remain the gold standard for diagnosing bloodstream infections [6]. The timely and accurate identification of pathogens and resistance markers detection is crucial to optimize patient outcomes and antimicrobial use [7]. However these technical developments call for improvements in other steps including the optimization of the pre-analytical parameters, the rapid start of incubation, reorganization (e.g., 24/7 blood culture processing), and the close involvement of antimicrobial stewardship teams to ensure the full use of these techniques in patient management. Indeed, continuous-monitoring blood culture systems and rapid diagnostics have reduced the time to results; however, the turnaround time still depends on laboratory organization [6,8,9]. Delays between culture positivity and diagnosis emphasize the importance of uninterrupted, 24/7 blood culture processing to avoid postponing critical therapeutic decisions.

Since 2012, a project aimed at improving sepsis management has been implemented at our hospital, Fondazione IRCCS Policlinico San Matteo in Pavia (Italy), with progressive steps introduced over time [10,11]. In 2015, blood culture incubators were placed in the emergency room, in the infectious diseases ward and in the intensive care units to reduce pre-incubation time [10,11]. Since 2019, an alert system to notify infectious disease consultants has enabled real-time consultations for preliminary BC results, improving antimicrobial stewardship programs. In 2020, a fast-track system was also introduced for BCs collected from critical patients with suspected sepsis and/or septic shock (identified as critical as MEWS ≥ 3 or NEWS-2 ≥ 5). These samples were flagged with a specific code—urgent blood cultures (Urg-BC)—to differentiate and prioritize them for rapid processing. Blood cultures were therefore managed 24/7 using an organizational model designed to provide a fast and preferential route for positive samples identified as ‘urgent’. This approach ensured diagnostic continuity and protected patient care, especially in septic shock, where time is critical. A dedicated team with structured shifts, including night coverage, a guaranteed uninterrupted around-the-clock workflow, and real-time response at any time.

On the basis of these organizational interventions, the present study aimed to evaluate the turnaround times (TATs) for Gram staining, microorganism identification, and resistance marker detection in BCs over the six-year period following the implementation of 24/7 laboratory organization and a fast-track, preferential diagnostic pathway dedicated to BCs from critical patients.

## 2. Results

### 2.1. Distribution of Positive Blood Cultures

**Figure 1:**
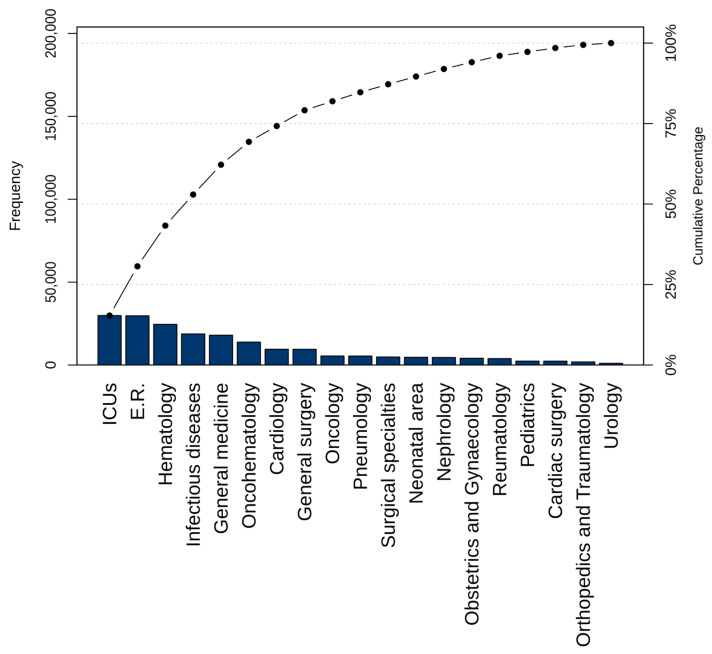
Pareto chart of requested blood culture bottles from 1 January 2019 to 31 December 2024, excluding COVID-19 period (1 January to 30 April 2020).

From 1 January 2019 to 31 December 2024 (excluding the COVID-19 period from 1 January to 30 April 2020), a total of 194,171 BCbs were processed from 23,508 patients who were hospitalized. Of these BCbs, 177,224 (91.27%) were Non-Urg-BCbs and 16,947 (8.73%) were Urg-BCbs. A total of 115,131 (59.29%) BCbs were collected during daytime, while the remaining 79,040 (40.70%) were collected during the night shifts. BCbs were collected mostly in the intensive care units (ICUs) and in the emergency room (ER), accounting for, respectively, 15.36% and 15.29% of the total, followed by the hematology (12.63%) and the infectious diseases (ID; 9.66%) wards (Figure 1). At least half of the BCbs collected during the night shift were from the ER (18.19%), ICUs (14.79%), hematology (12.08%), and general medicine (9.63%).

Urg-BCbs were collected mainly from patients admitted to the ICU and ER, at 34.44% and 32.22% respectively. The remaining 33.44% of the Urg-BCbs were collected from different wards.

**Figure 2:**
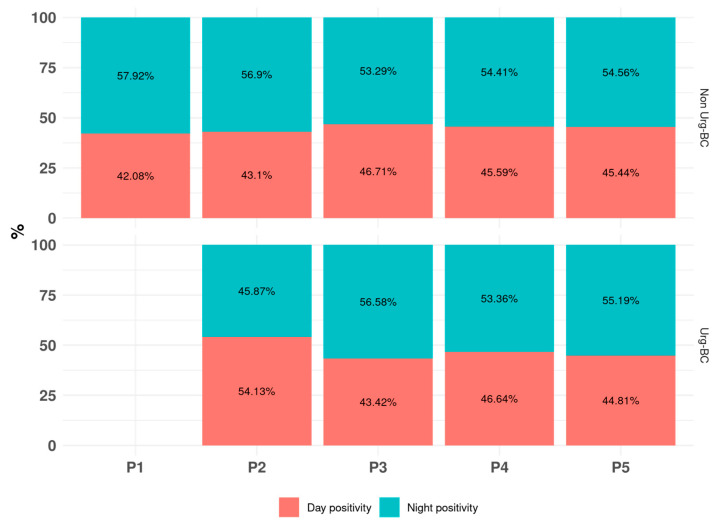
Percentage of positive blood cultures during day and night, stratified by period (P1 to P5) and type of blood culture (Non-Urg-BC and Urg-BC).

During the study period, 25,858 (13.31%) BCbs tested positive, of which 23,109 were Non-Urg-BCbs and 2749 were Urg-BCbs (13.04% and 16.22%, respectively, of the total number of requested Non-Urg-BCbs and Urg-BCbs, *p* < 0.001). The BCbs were flagged positive a median of 14.09 [Q1–Q3: 10.29–19.38] h after incubation, with a BCb contamination rate of 5.53%. The first encounter accounted for 10,340 of the BCbs (9086 Non-Urg-BCbs and 1254 Urg-BCbs). The percentages of BCbs that became positive during day and night were comparable among the five periods and did not significantly differ (Figure 2). The median percentage of BCbs flagged positive during the night was 54.56% [53.36–56.58] and 45.35% [44.50–45.71] during the day.

### 2.2. Gram Stain Turnaround Time

**Figure 3:**
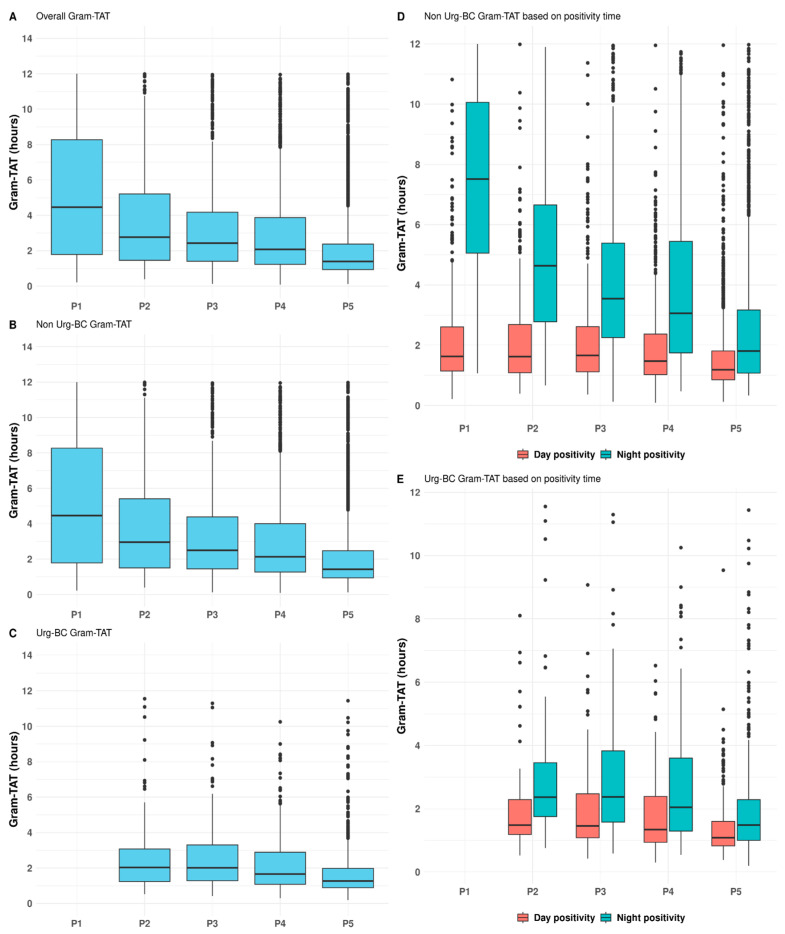
Gram stain TAT during the five periods, divided by overall Gram stain TAT (**A**), Non-Urg-BC stain TAT (**B**), Urg-BC Gram stain TAT (**C**), Non-Urg-BC Gram stain TAT based on positivity time (day/night) (**D**), Urg-BC Gram stain TAT based on positivity time (**E**).

In 2019 (period P1), when the CML operated 12 h per day, the median time from BCb positivity to Gram stain result (Gram-TAT) was 4.46 [Q1–Q3: 1.79–8.27] h. However, following the introduction of the night shift and transition to a 24/7 operational model (period P2), the overall median Gram-TAT decreased significantly. Specifically, it declined to 2.43 [1.40–4.18] h in P3 and further to 1.40 [0.94–2.38] h in P5 (Figure 3A and Table 1). This decreasing trend in Gram-TAT was statistically significant during the study period (*p* < 0.001), with an r_rb_ = 0.42 [95% CI: 0.38–0.45] when comparing P2 to P5. Notably for BCbs that were flagged positive during night, the median Gram-TAT was 7.51 [5.06–10.06] h in P1 and reached 1.74 [1.06–3.06] h in P5 (*p* < 0.001).

For Non-Urg-BCbs, the median hours for Gram-TAT decreased from 4.46 in P1 to 2.5 h in P3 (*p* < 0.001; r_rb_ = 0.56 [95% CI: 0.53–0.58]). As shown in Figure 3B, the Gram-TAT continued to decrease significantly in P4 and P5, reaching 1.43 h in the validation period P5. The Gram-TAT of the Non-Urg-BCbs that became positive during the night decreased from 7.51 [5.06–10.06] in P1 to 1.81 [1.08–3.17] h in P5, with the decreasing trend being statistically significant (*p* < 0.001), as shown in Figure 3D.

For the Urg-BCbs during the study periods (P1 excluded), the median Gram-TAT was 1.44 [0.97–2.39], which was significantly lower than the 1.73 [1.05–3.18] h for Non-Urg-BC (*p* < 0.001). In P2, the Gram-TAT was 2.04 [1.24–3.08] and decreased to 1.27 [0.89–1.98] h in the validation period, P5 (*p* < 0.001; r_rb_ = 0.34 [95% CI: 0.24–0.44]) (Figure 3C). The Gram-TAT of the Urg-BCbs that became positive during the night decreased from 2.37 [1.72–3.45] h in P2 to 1.48 [1.00–2.29] h in P5, with the decreasing trend being statistically significant (*p* < 0.001) (Figure 3E).

### 2.3. Gram Staining Results

**Figure 4:**
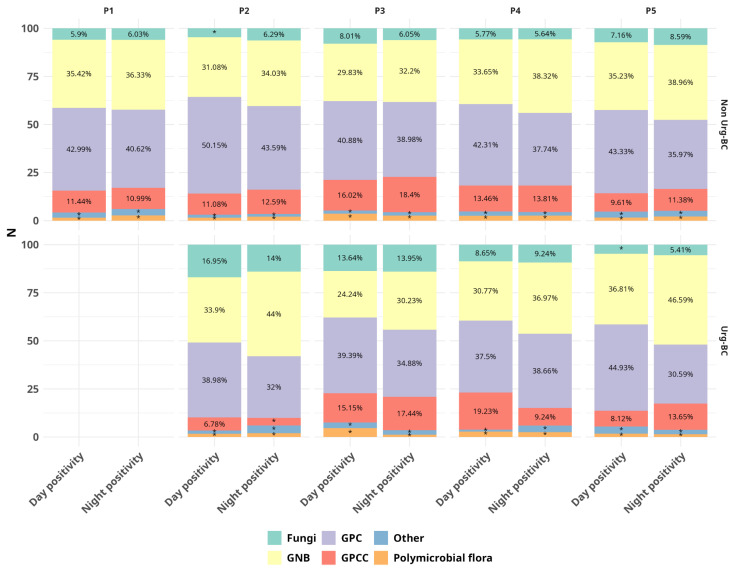
Gram stain classification of positive blood cultures during day and night, stratified by period (P1 to P5) and type of blood culture (Non-Urg-BC and Urg-BC). GNB = Gram-negative bacilli, GPC = Gram-positive cocci, GPCC = Gram positive cocci in chains. Values below 5% are indicated with *.

Considering the total positive BCbs, Gram-positive cocci (GPC) accounted for 39.9% (including BC contaminations), followed by Gram-negative bacilli (GNB) (36.32%), Gram-positive cocci in chains (GPCC) (11.88%), fungi (7.08%) and polymicrobial flora (2.15%) (Figure 4). In terms of the Gram stain result, no statistically significant difference was observed in the percentage of fungi, Gram-positive cocci (GPC) and polymicrobial flora among the five periods.

The GPC percentage was 40.37% for the Non-Urg-BCbs and 37.08% for the Urg-BCbs (*p* = 0.03); the GNB percentage was 35.99% for the Non-Urg-BCbs and 38.68% for the Urg-BCbs (*p* > 0.05); the GPCC percentage was 11.89% for the Non-Urg-BCbs and 11.80% for the Urg-BCbs (*p* > 0.05). Fungi accounted for 6.99% and 7.74% of the Non-Urg-BCbs and Urg-BCbs, respectively (*p* > 0.05). Polymicrobial flora accounted for 2.18% and 1.91% of the Non-Urg-BCbs and Urg-BCbs, respectively (*p* > 0.05).

### 2.4. Microorganism Identification Turnaround Time

In cases where rapid identification was performed, the overall ID-TAT significantly decreased from 5.75 (3.04–9.60) h in P1 to 2.35 (1.76–3.42) h in P5 (P1 vs. P5: *p* < 0.05; r_rb_ = 0.59 [95% CI: 0.54–0.63]), as shown in Table 1. In particular for BCbs that were flagged positive during the night, the median ID-TAT was 8.71 [6.28–11.13] h in P1 and 2.35 [1.68–4.00] h in P5 (*p* < 0.001).

For the Non-Urg-BCbs, the median ID-TAT in P1 was 5.75 [3.04–9.60], while, in P5, it was 2.37 [1.77–3.53] h, which was a statistically significant decrease (*p* < 0.001; r_rb_ = 0.55 [95% CI: 0.50–0.60]). The decreasing trend in the ID-TAT was especially observed for BCbs that were flagged positive during the night, changing from 8.71 [6.28–11.13] in P1 to 2.41 [1.70–4.21] h in P5 (*p* < 0.001). For those flagged positive during daytime, the median time remained stable between 2.73 [2.05–3.55] in P1 and 2.37 [1.87–3.05] h in P5.

**Figure 5:**
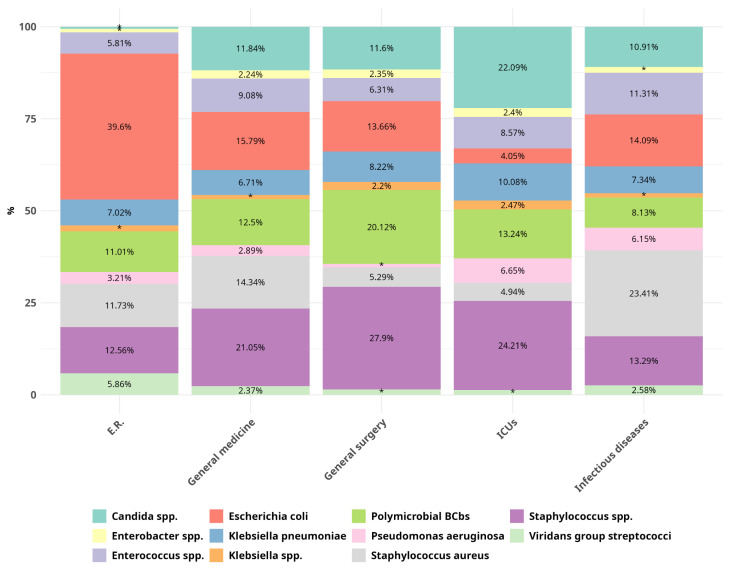
Percentage of the microorganisms most frequently isolated from BCs in five representative wards. Values below 2% are indicated with *.

For the Urg-BCbs, the ID-TAT changed from 2.72 [2.15–4.42] in P2 to 2.21 [1.60–3.18] h in P5 (*p* < 0.001 for the trend). Moreover, a significant decrease was observed for the Urg-BCbs that were flagged positive during the night in P5 compared to all previous periods (*p* < 0.001 for the trend). Conversely, ID-TAT remained unchanged for the Urg-BCbs flagged positive during the day. The microorganisms isolated from positive blood cultures are shown in Table 2 and Figure 5. From this point onward, the BCBs identified as contaminants were excluded from the analysis.

### 2.5. Microorganism Identification Results

GPC were the most frequently isolated bacteria, with *Staphylococcus* spp. accounting for 18.82% of the BCbs, including *Staphylococcus aureus* (9.06%) and *Enterococcus* spp. representing 6.8%. Gram-negative bacteria accounted for 38.47% of all isolates, with *Escherichia coli* being the most prevalent species (16.26%). Fungi were isolated in 7.67% of the cases and polymicrobial flora in 10.72% (Table 2 and Figure 5). 

In the main five medical areas (ER, general medicine, general surgery, ICUs, and ID) in our hospital, *E. coli* (39.60%) was the most frequently isolated microorganism in the ER, *Candida* spp. in the ICU (22.09%), *S. aureus* in the infectious diseases ward (23.41%), and polymicrobial infections in general surgery (20.19%) (Figure 5). Notably, *Streptococcus pneumoniae* was isolated more frequently from Urg-BCbs (1.28%) than from Non-Urg-BCbs (0.89%). Overall, BCbs positive for bacteria that can cause meningitis (i.e., *S*. *pneumoniae*, *N. meningitidis*, and *Haemophilus influenzae*) were equally positive during night and day.

### 2.6. Resistance Markers Turnaround Time

The overall Marker-TAT significantly decreased from 6.97 [3.63–11.05] in P1 to 2.68 [1.94–3.97] h in P5 (*p* < 0.05; r_rb_ = 0.60 [95% CI: 0.51–0.67]), see Table 2. For the Non-Urg-BCbs, the median Marker-TAT significantly decreased from 6.97 [3.63–11.05] in P1 to 2.71 [1.98–4.07] h in P5 (*p* < 0.05; r_rb_ = 0.58 [95% CI: 0.49–0.65]). Moreover, for Non-Urg-BCbs that were flagged positive during the night, Marker-TAT significantly decreased from 7.18 [5.54–8.63] in P2 to 2.85 [1.86–5.53] h in P5. For the Urg-BCbs, Marker-TAT decreased from 4.90 [2.30–7.26] in P2 to 2.51 [1.79–3.61] in P5; however, no statistical significance was observed.

### 2.7. Resistance Marker Identification

**Figure 6:**
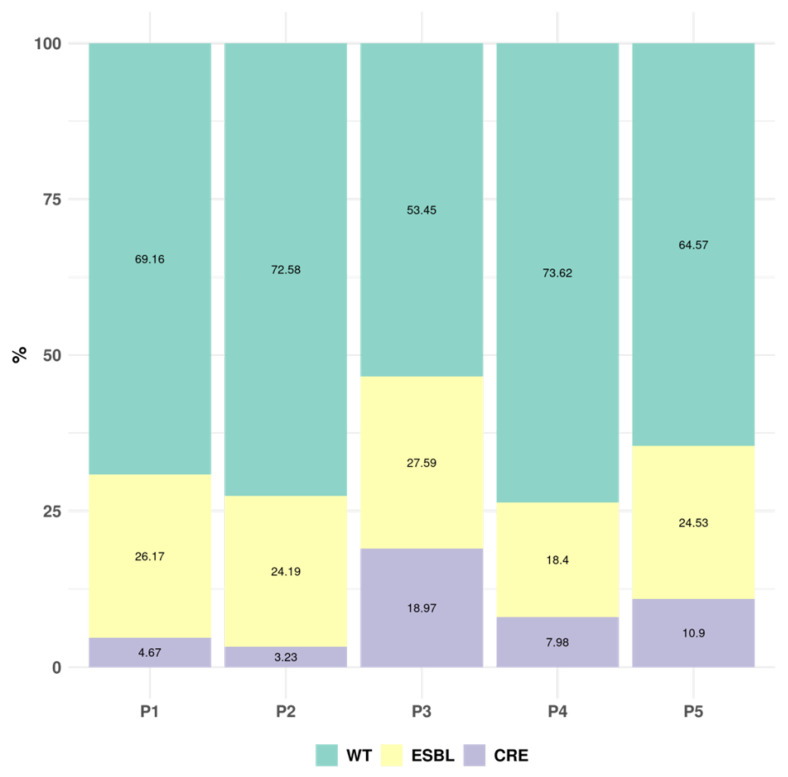
Percentage of blood cultures with no markers (wildtype), extended-spectrum beta-lactamase-producing *Enterobacterales* (ESBL) and carbapenemase-producing Gram negative bacilli (CRE).

Finally, considering the GNB resistance markers in the five periods, the percentage of extended-spectrum beta-lactamase (ESBL)-producing *Enterobacteriaceae* was stable over time (18.40–27.59%), while carbapenem-resistant *Enterobacterales* (CRE) increased after the first wave of the COVID-19 pandemic from 3.23% in P2, reaching 18.97% in P3 and stabilizing in P4 and P5 (7.98% and 10.9%) (Figure 6).

## 3. Discussion

The prompt recognition and treatment of sepsis are essential, as mortality associated with septic shock increases by approximately 7% for every hour of delay in initiating appropriate antimicrobial therapy [12,13]. Rapid microbiological diagnosis, encompassing pre-analytical, analytical, and post-analytical, must be efficient to enable timely identification and response [5,14,15].

Starting in May 2020, the clinical microbiology laboratory was reorganized with expanded staff capacity and the introduction of night shifts, enabling the 24/7 processing of BCs. From that date onward, clinicians have also been able to request urgent BCs, which were identified with a specific code to be recognized and processed with priority, in order to accelerate the diagnosis for patients with sepsis at higher risk of death. This reorganization and the implementation of urgent BC processing allowed a reduction in the analytical turnaround time (TAT) for BCs, measured as Gram-TAT, ID-TAT, and Marker-TAT, during both daytime and night-time hours.

During the study period, approximately 13% of the collected BCbs were positive, and over half of these (54%) showed positivity during night-time hours. At the start of the observation period (P1), the median Gram-TAT for BCbs flagged positive during the night was approximately 8 h. By period P5 (P5), this had decreased to 1.27 h for BCbs classified as urgent and 1.48 h for non-urgent BCbs, values that were comparable to those observed in period 4 (P4), indicating consistency of performance over time.

The Gram staining results play a crucial role in the management of patients with sepsis and can be associated with decreased patient mortality [16,17,18]. In particular, the association between prompt Gram stain reporting and reduced mortality is mainly mediated by faster clinical decision-making at the bedside. An early Gram stain can immediately suggest the likely pathogen group and morphology, allowing clinicians to quickly refine empirical therapy (escalation/de-escalation). This earlier guidance can reduce the time to the administration of appropriate antimicrobials, improve patient management, and support antimicrobial stewardship. In this study, the improved Gram stain turnaround time achieved through the 24/7 workflow likely contributed to the faster communication of actionable microbiological data. Cattoir and colleagues evidenced that about one-third of positive blood cultures led to antibiotic initiation or switch based on the Gram stain result, mostly due to the presence of Gram-positive stains or yeasts [19]. In our study, Gram-positive bacteria accounted for 43.13% of the total, followed by Gram-negative bacteria (38.47%), and yeasts (7.67%).

Recent advances in rapid microorganism identification and antimicrobial susceptibility testing have substantially reduced TATs [20,21,22,23]. By the end of period 5, microorganism identification and major resistance mechanisms were available within a median time of less than 3 h after blood culture positivity, corresponding to a median of 17 h from blood culture collection. During this time, critical bacteria that can cause meningitis (e.g., *Neisseria meningitidis*, *Streptococcus pneumoniae*, and *Haemophilus influenzae*) and yeasts such as *Candida* spp. were also identified, underlining the need for a fast processing pathway for BCs and the communication of the results at all hours [24,25] (see Table 2). This rapid workflow is particularly relevant given the increasing prevalence of multidrug-resistant organisms, especially among Gram-negative bacteria in the post-COVID-19 era [26,27,28].

Given that clinical decision-making relies on laboratory data, enhancing the speed and accuracy of these results can influence treatment pathways and patient outcomes [20]. Shorter intervals between BC collection and communication of results have consistently been linked to reduced mortality [16,29]. Earlier microbiological information allows the faster initiation or optimization of targeted antimicrobial therapy, more rapid de-escalation from broad-spectrum empirical treatment, and quicker clinical decisions in patients with sepsis or bloodstream infection. Our data showed a process improvement, with a marked reduction in turnaround times after implementation of the 24/7 workflow and fast-track pathway, with Gram stain, identification, and resistance-marker reporting becoming substantially faster over time. As the onset of sepsis during night shifts is associated with longer antimicrobial therapy delays and higher mortality compared with the day shifts [30,31], efforts were made to prioritize the processing of positive BCbs during night shifts, especially for Urg-BCs requested for critical patients.

Urg-BCs represented roughly 10% of all BCs collected in the study period. Urg-BCs were mainly collected from the ER and ICU, and their prevalence remained stable over time. The Urg-BC positivity rate was higher than that of non-Urg-BCs, at 16% versus 13%, respectively. For example, *S. pneumoniae*-positive BCs were more frequent among Urg-BCs. The Urg-BC concept can be seen as a form of tailored medicine: it signals to CML personnel the critical status of a patient, allowing them to prioritize processing and deliver rapid results. The timely communication and active, rapid action by clinicians are essential for effective sepsis management [32]. At the beginning of this reorganization, only Urg-BCs were processed during night shifts; however, in P5, all blood cultures were processed overnight, resulting in a reduction in TATs for all BCs.

This study has a potential limitation due to the lack of available outcome data, such as mortality and length of stay, since these parameters were not systematically recorded during the study period and therefore could not be included in the present analysis. These operational improvements can be directly integrated into hospital antimicrobial stewardship programs, where rapid laboratory data are used to trigger structured review and the adjustment of antibiotic regimens, thereby enhancing both patient-level outcomes and antimicrobial resistance control. In the future, this model could be combined with other emerging rapid diagnostic technologies, offering an around-the-clock framework to enhance the management of sepsis and bloodstream infections and to strengthen the quality of care and antimicrobial stewardship programs.

## 4. Materials and Methods

The Fondazione IRCCS Policlinico San Matteo (Pavia, Italy) is a 900-bed tertiary-care teaching hospital recording about 28.000 hospitalizations per year. When sepsis/bacteremia was suspected in adult patients, two or three sets of blood culture bottles (BCbs) were collected, as recommended by CLSI guidelines [2,33]. Each specimen should contain at least 10 mL of blood, resulting in a total recommended volume of 40–60 mL.

### 4.1. Definitions

Blood culture (BC) was defined as two or three sets of blood culture bottles (BCbs), including aerobic and anaerobic bottles, collected from the same patient on the same day.

Urgent blood culture (Urg-BC) was defined as BCbs collected from patients identified as critical as MEWS ≥ 3 or NEWS-2 ≥ 5 [34,35]. These BCs were labeled with a specific code when the sample was accepted into the laboratory’s operating system. Software connected to the laboratory information system (LIS) stored the information and alerted the personnel on duty through an automated phone call in case of BC positivity.

Non-urgent blood cultures (Non-Urg-BC) were BCbs collected for non-critical patients.

Time to detection (TTD) was the time elapsed between BCb incubation and positivity.

First encounter was the BCb with lower TTD among the BCbs collected from each patient within the same day considering Gram stain type.

Gram stain turnaround time (Gram-TAT) was the time elapsed between the positivity of BCb and the Gram staining result.

Microorganism identification turnaround time (ID-TAT) was the time elapsed between BCb positivity and microorganism identification.

Resistance marker turnaround time (Marker-TAT) was the time elapsed between the BCb positivity and the identification of antibiotic resistance markers.

BCb contamination: Coagulase-negative staphylococci, aerobic and anaerobic diphtheroids, *Micrococcus* spp., *Bacillus* spp., and viridans streptococci were considered contaminants when a single BCb was positive. The isolate was reported as a probable contaminant, and susceptibility testing was not performed [2,36].

### 4.2. Study Design

**Figure 7:**
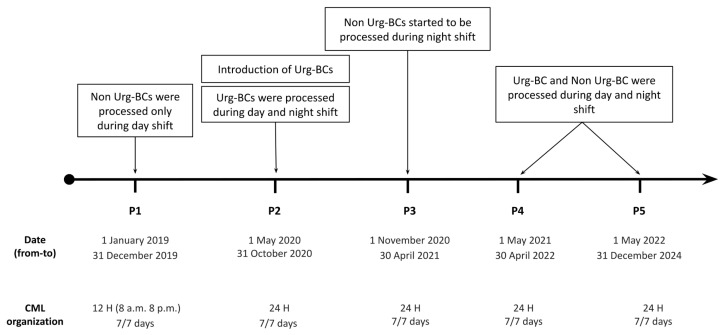
Schematic timeline of study periods from period 1 (P1) to period 5 (P5) and relative clinical microbiology laboratory organization.

This retrospective study considered BCbs collected from 1 January 2019 to 31 December 2024, excluding those from the first wave of the COVID-19 pandemic (1 January to 30 April 2020), as routine laboratory workflows were significantly disrupted during this period. Starting from January 2020, a fast-track and preferential diagnostic pathway dedicated to Urg-BC was introduced. In the same period, all personnel—including those from the virology, mycology, and parasitology units—were trained to handle positive BCbs during both day and night shifts. Throughout the entire study period, BCbs were incubated 24/7. Positive BCbs were processed during the operating hours of the clinical microbiology laboratory (CML), which changed over the course of this study, as detailed in Figure 7.

In detail, positive BCbs were processed during the operating hours of the clinical microbiology laboratory (CML) following this scheme:

- Period 1 (P1): from 1 January 2019 to 31 December 2019, baseline period. CML organization was 7 working days a week, operating from 8 am to 8 pm.
- Period 2 (P2): from 1 May 2020 to 31 October 2020. Introduction of night shifts in CML organization and of Urg-BC. CML organization was 24/7. All BCbs were processed during the daytime (from 8 am to 8 pm). Urg-BCbs were also processed during the night shift (from 8 pm to 8 am).
- Period 3 (P3): from 1 November 2020 to 30 April 2021. BCb processing was implemented during the night shift. CML operated 24/7. All BCbs were processed during the daytime; Urg-BCbs were also processed during the night shift. Non-Urg-BCb processing was gradually introduced during the night shift as personnel training was implemented.
- Period 4 (P4): from 1 May 2021 to 30 April 2022. Integration of complete activity into the overall process of improvement in sepsis diagnosis. All personnel completed the training, enabling all BCbs (Urg-BCbs and Non-Urg-BCbs) to be handled equally during both day and night shifts.
- Period 5 (P5): from 1 May 2022 to 31 December 2024, validation period. In this period we evaluated whether the results of the previous periods were maintained over time.

### 4.3. Laboratory Workflow

BCbs were incubated within 2 h of collection at BACTEC FX (Becton Dickinson and Company, Franklin Lakes, NJ, USA), located in the CML, or in BACTEC FX 40, located in the Emergency Room, Infectious Diseases ward and Intensive Care Unit. When a BCb flagged positive, Gram-staining was immediately performed; the result was recorded in the laboratory information system (LIS) and promptly communicated by phone to the ward physician. Concurrently, the BCb was plated on appropriate agar media, as indicated by internal procedures.

Rapid identification was guided by the Gram stain result, in accordance with laboratory protocols. In case of Gram-negative bacilli (GNB), Gram-positive bacilli (GPB) and Gram-positive cocci in chains (GPCC), identification was achieved by Matrix Assisted Laser Desorption Ionization-Time of Flight (MALDI-ToF) mass spectrometry (Bruker Daltonik, Bremen, Germany). In the case of Gram-positive cocci (GPC), the coagulase test was used to identify Staphylococcus aureus.

When polymicrobial flora or yeasts were observed, a multiplex PCR syndromic panel (FilmArray Blood Culture-2 Panel BioFire diagnostics LLC, Salt Lake City, UT, USA) was used in order to identify the microorganisms involved and their resistance marker.

Following rapid identification, resistance markers for *Enterobacterales* were assessed using either a molecular Xpert^®^ CARBA-R assay (Cepheid, Sunnyvale, CA, USA) or an immunochromatographic test for carbapenemase production (NG-Test CARBA5, NG Biotech Laboratoire, Guipry, France). For the detection of CTX-M-type extended-spectrum β-lactamases (ESBLs), an NG-Test^®^ CTX-M Multi (NG Biotech Laboratoire, Guipry, France) was used. In the case of Enterococci, Molecular Xpert^®^ vanA/vanB (Cepheid, USA) was used to detect vancomycin resistance. For Staphylococci, methicillin resistance was evaluated using a molecular MRSA/SA Blood Culture assay (Cepheid, USA).

### 4.4. Data Processing and Statistical Analysis

All data used in this study were extracted from the LIS, which recorded all BCbs submitted to the CML. Both positive and negative BC bottles were included in the analysis. First-encounter BCb was considered in the analysis, and BCbs identified as contamination according to CLSI guidelines were excluded [2,36].

Continuous variables (Gram-TAT, ID-TAT, Marker-TAT) are reported as medians with interquartile ranges (Q1–Q3). Comparisons of variable distributions across different study periods were performed using the Kruskal–Wallis test, followed by Dunn’s post hoc test with Bonferroni’s correction. To estimate effect size, we performed the rank eta-squared test followed by the Rank-Biserial Correlation (r_rb_) to find differences between two groups.

To assess whether there was a monotonic decreasing trend in Gram-TAT and ID-TAT across the five consecutive periods, we applied Kendall’s rank correlation test (alternative hypothesis “less”) with periods treated as ordered time points. Within each period, the comparison of Non-Urg-BC and Urg-BC regarding continuous variables was performed using the Wilcoxon rank-sum test.

Categorical variables are presented as absolute counts and percentages. Proportions were compared using the chi-squared (χ^2^) test or the binomial test when only two categories were present. To assess whether the proportions of each Gram stain result varied across periods, we performed a proportion test. The significance level was assumed to be 0.05.

## 5. Conclusions

The reorganization of our clinical microbiology laboratory led to the establishment of a fully integrated, around-the-clock system for processing BCs. With this rapid and preferential workflow for positive blood cultures designated as ‘urgent’, rapid communication protocols, and advanced diagnostic technologies, we have enhanced our hospital’s capacity to respond to sepsis both promptly and effectively.

This model exemplifies how tailored and precision microbiology, supported by strong interdisciplinary collaboration and effective communication, can enhance earlier targeted antimicrobial treatment. This approach not only reinforces antimicrobial stewardship but also lays the groundwork for future innovation in the real-time diagnosis of life-threatening infections.

## Figures and Tables

**Table 1:** Summary of time for Gram-TAT, ID-TAT, and Marker-TAT for overall and type of blood culture (Non-Urg-BC and Urg-BC). Median time [Q1–Q3] is indicated in hours. Arrows indicate the trend.

	Gram-TAT			ID-TAT			Marker-TAT		
Period	Non-Urg-BC	Urg-BC	Overall	Non-Urg-BC	Urg-BC	Overall	Non-Urg-BC	Urg-BC	Overall
P1	4.46 [1.79–8.27]		4.46 [1.79–8.27]	5.75 [3.04–9.59]		5.75 [3.04–9.59]	6.97 [3.63–11.05]		6.97 [3.63–11.05]
P2	2.96 [1.51–5.41]	2.04 [1.24–3.08]	2.77 [1.46–5.21]	4.05 [2.54–6.77]	2.72 [2.15–4.42]	3.91 [2.47–6.61]	5.71 [3.39–7.73]	4.91 [2.30–7.26]	5.59 [3.35–7.73]
P3	2.5 [1.45–4.38]	2.01 [1.28–3.30]	2.43 [1.40–4.18]	3.49 [2.43–5.50]	3.06 [2.57–5.08]	3.41 [2.44–5.50]	3.69 [2.56–6.23]	2.96 [2.33–5.39]	3.61 [2.45–5.84]
P4	2.13 [1.27–4.00]	1.66 [1.09–2.89]	2.08 [1.24–3.87]	2.86 [2.12–4.89]	2.76 [2.07–4.41]	2.85 [2.11–4.83]	4.03 [2.48–6.49]	3.05 [2.43–3.94]	3.70 [2.44–6.15]
P5	1.43 [0.94–2.48]	1.27 [0.89–1.98]	1.40 [0.94–2.38]	2.37 [1.77–3.53]	2.21 [1.60–3.18]	2.35 [1.76–3.42]	2.71 [1.98–4.07]	2.51 [1.79–3.61]	2.68 [1.94–3.97]
Significant trend	↓ (*p* < 0.001)	↓ (*p* < 0.001)	↓ (*p* < 0.001)	↓ (*p* < 0.001)	↓ (*p* < 0.001)	↓ (*p* < 0.001)	↓ (*p* < 0.001)	↓ (*p* = 0.03)	↓ (*p* < 0.001)

**Table 2:** Microorganisms isolated from Non-Urg-BCbs and Urg-BCbs.

	Overall		Non-Urg-BC		Urg-BC	
	N	%/tot Pos BCs (9176)	N	%/tot Pos BCs (8078)	N	%/tot Pos BCs (1098)
*Escherichia coli*	1492	16.26%	1284	15.90%	208	18.94%
*Klebsiella pneumoniae*	609	6.64%	534	6.61%	75	6.83%
*Pseudomonas aeruginosa*	331	3.61%	288	3.57%	43	3.92%
*Enterobacter* spp.	168	1.83%	144	1.78%	24	2.19%
*Klebsiella* spp.	144	1.57%	129	1.6%	15	1.37%
*Salmonella* spp.	24	0.26%	20	0.25%	4	0.36%
*H. influenzae/N. meningitidis*	14	0.15%	14	0.17%	0	-
Anaerobic Gram-negative	102	1.11%	93	1.15%	9	0.82%
Other Gram-negative	646	7.04%	565	6.99%	81	7.38%
*Staphylococcus* spp.	1727	18.82%	1552	19.21%	175	15.94%
*Staphylococcus aureus*	831	9.06%	744	9.21%	87	7.92%
*Enterococcus* spp.	624	6.8%	546	6.76%	78	7.1%
*Viridans group streptococci*	290	3.16%	261	3.23%	29	2.64%
*Streptococcus pneumoniae*	86	0.94%	72	0.89%	14	1.28%
*Streptococcus agalactiae*	31	0.34%	24	0.3%	7	0.64%
*Streptococcus pyogenes*	22	0.24%	16	0.2%	6	0.55%
*Listeria* spp.	13	0.14%	10	0.12%	3	0.27%
Anaerobic Gram-positive	74	0.81%	64	0.79%	10	0.91%
Other Gram-positive	260	2.83%	220	2.72%	40	3.64%
*Candida* spp.	704	7.67%	609	7.54%	95	8.65%
Polymicrobial BCbs	984	10.72%	889	11.01%	95	8.65%

## Data Availability

The original contributions presented in this study are included in the article. Further inquiries can be directed to the corresponding author.

## References

[1] Van Heuverswyn J., Valik J.K., Desirée van der Werff S., Hedberg P., Giske C., Nauclér P. (2023). Association Between Time to Appropriate Antimicrobial Treatment and 30-day Mortality in Patients With Bloodstream Infections: A Retrospective Cohort Study. Clin. Infect. Dis..

[2] Clinical Laboratory Standards Institute (CLSI) (2022). Principles and Procedures for Blood Cultures.

[3] Evans L., Rhodes A., Alhazzani W., Antonelli M., Coopersmith C.M., French C., Machado F.R., Mcintyre L., Ostermann M., Prescott H.C. (2021). Surviving sepsis campaign: International guidelines for management of sepsis and septic shock 2021. Crit. Care Med..

[4] King J., Chenoweth C.E., England P.C., Heiler A., Kenes M.T., Raghavendran K., Wood W., Zhou S., Mack M., Wesorick D. (2023). Early Recognition and Initial Management of Sepsis in Adult Patients.

[5] Lockwood A.M., Perez K.K., Musick W.L., Ikwuagwu J.O., Attia E., Fasoranti O.O., Cernoch P.L., Olsen R.J., Musser J.M. (2016). Integrating rapid diagnostics and antimicrobial stewardship in two community hospitals improved process measures and antibiotic adjustment time. Infect. Control Hosp. Epidemiol..

[6] Lamy B., Dargère S., Arendrup M.C., Parienti J.-J., Tattevin P. (2016). How to Optimize the Use of Blood Cultures for the Diagnosis of Bloodstream Infections? A State-of-the Art. Front. Microbiol..

[7] Simner P.J., Dien Bard J., Doern C., Kristie Johnson J., Westblade L., Yenokyan G., Patel R., Hanson K.E. (2023). Antibacterial Resistance Leadership Group. Reporting of Antimicrobial Resistance from Blood Cultures, an Antibacterial Resistance Leadership Group Survey Summary: Resistance Marker Reporting Practices from Positive Blood Cultures. Clin. Infect. Dis..

[8] Tabak Y.P., Vankeepuram L., Ye G., Jeffers K., Gupta V., Murray P.R. (2018). Blood culture turnaround time in U.S. acute care hospitals and implications for laboratory process optimization. J. Clin. Microbiol..

[9] Lamy B., Sundqvist M., Idelevich E.A. (2020). ESCMID Study Group for Bloodstream Infections, Endocarditis and Sepsis (ESGBIES). Bloodstream infections—Standard and progress in pathogen diagnostics. Clin. Microbiol. Infect..

[10] Seminari E., Colaneri M., Corbella M., De Silvestri A., Muzzi A., Perlini S., Martino I.F., Marvulli L.N., Arcuri A., Maffezzoni M. (2023). Reduction of BSI associated mortality after a sepsis project implementation in the ER of a tertiary referral hospital. Sci. Rep..

[11] Mariani B., Corbella M., Seminari E., Sacco L., Cambieri P., Capra Marzani F., Martino I.F., Bressan M.A., Muzzi A., Marena C. (2018). Evaluation of a model to improve collection of blood cultures in patients with sepsis in the emergency room. Eur. J. Clin. Microbiol. Infect. Dis..

[12] Kumar A., Roberts D., Wood K.E., Light B., Parrillo J.E., Sharma S., Suppes R., Feinstein D., Zanotti S., Taiberg L. (2006). Duration of hypotension before initiation of effective antimicrobial therapy is the critical determinant of survival in human septic shock. Crit. Care Med..

[13] Kahn J.M., Davis B.S., Yabes J.G., Chang C.C.H., Chong D.H., Hershey T.B., Martsolf G.R., Angus D.C. (2019). Association Between State-Mandated Protocolized Sepsis Care and In-hospital Mortality Among Adults With Sepsis. JAMA.

[14] Morquin D., Lejeune J., Agostini C., Godreuil S., Reynes J., Le Moing V., Lamy B. (2024). Time is of the essence: Achieving prompt and effective antimicrobial therapy of bloodstream infection with advanced hospital information systems. Clin. Infect. Dis..

[15] Venturelli C., Righi E., Borsari L., Aggazzotti G., Busani S., Mussini C., Rumpianesi F., Rossolini G.M., Girardis M. (2017). Impact of Pre-Analytical Time on the Recovery of Pathogens from Blood Cultures: Results from a Large Retrospective Survey. PLoS ONE.

[16] Barenfanger J., Graham D.R., Kolluri L., Sangwan G., Lawhorn J., Drake C.A., Verhulst S.J., Peterson R., Moja L.B., Ertmoed M.M. (2008). Decreased mortality associated with prompt Gram staining of blood cultures. Am. J. Clin. Pathol..

[17] Perez K.K., Olsen R.J., Musick W.L., Cernoch P.L., Davis J.R., Peterson L.E., Musser J.M. (2014). Integrating rapid diagnostics and antimicrobial stewardship improves outcomes in patients with antibiotic-resistant Gram-negative bacteremia. J. Infect..

[18] Messacar K., Parker S.K., Todd J.K., Dominguez S.R. (2017). Implementation of rapid molecular infectious disease diagnostics: The role of diagnostic and antimicrobial stewardship. J. Clin. Microbiol..

[19] Cattoir L., Coorevits L., Leroux-Roels I., Claeys G., Verhasselt B., Boelens J. (2018). Improving timelines in reporting results from positive blood cultures: Simulation of impact of rapid identification on therapy on a real-life cohort. Eur. J. Clin. Microbiol. Infect. Dis..

[20] Antonios K., Croxatto A., Culbreath K. (2021). Current state of laboratory automation in clinical microbiology laboratory. Clin. Chem..

[21] Banerjee R., Komarow L., Virk A., Rajapakse N., Schuetz A.N., Dylla B., Earley M., Lok J., Kohner P., Ihde S. (2021). Randomized Trial Evaluating Clinical Impact of RAPid IDentification and Susceptibility Testing for Gram-negative Bacteremia: RAPIDS-GN. Clin. Infect. Dis..

[22] Yuceel-Timur I., Thierry E., Chainier D., Ndao I., Labrousse M., Grélaud C., Bala Y., Barraud O. (2024). Retrospective evaluation of rapid genotypic ID and phenotypic AST systems on positive blood culture turnaround time and simulated potential impacts on bloodstream infection management. J. Antimicrob. Chemother..

[23] Schneiderhan W., Grundt A., Wörner S., Findeisen P., Neumaier M. (2013). Work flow analysis of around-the-clock processing of blood culture samples and integrated MALDI-TOF mass spectrometry analysis for the diagnosis of bloodstream infections. Clin. Chem..

[24] Deghmane A.-E., Taha S., Taha M.-K. (2022). Global epidemiology and changing clinical presentations of invasive meningococcal disease: A narrative review. Infect. Dis..

[25] Pappas P.G., Kauffman C.A., Andes D.R., Clancy C.J., Marr K.A., Ostrosky-Zeichner L., Reboli A.C., Schuster M.G., Vazquez J.A., Walsh T.J. (2016). Clinical practice guideline for the management of candidiasis: 2016 update by the infectious diseases society of america. Clin. Infect. Dis..

[26] Fasciana T., Antonelli A., Bianco G., Lombardo D., Codda G., Roscetto E., Perez M., Lipari D., Arrigo I., Galia E. (2023). Multicenter study on the prevalence of colonization due to carbapenem-resistant *Enterobacterales* strains before and during the first year of COVID-19, Italy 2018–2020. Front. Public Health.

[27] European Centre for Disease Prevention and Control (2023). Antimicrobial Resistance in the EU/EEA (EARS-Net)-Annual Epidemiological Report 2022.

[28] Kumar A., Ellis P., Arabi Y., Roberts D., Light B., Parrillo J.E., Dodek P., Wood G., Kumar A., Simon D. (2009). Initiation of inappropriate antimicrobial therapy results in a fivefold reduction of survival in human septic shock. Chest.

[29] French K., Evans J., Tanner H., Gossain S., Hussain A. (2016). The Clinical Impact of Rapid, Direct MALDI-ToF Identification of Bacteria from Positive Blood Cultures. PLoS ONE.

[30] Ginestra J.C., Kohn R., Hubbard R.A., Auriemma C.L., Patel M.S., Anesi G.L., Kerlin M.P., Weissman G.E. (2023). Association of Time of Day with Delays in Antimicrobial Initiation among Ward Patients with Hospital-Onset Sepsis. Ann. Am. Thorac. Soc..

[31] Lyons P.G., Hough C.L. (2023). Antimicrobials in sepsis: Time to pay attention to when delays happen. Ann. Am. Thorac. Soc..

[32] Halstead F.D., Pinjuh G., Antonacci G., Proudlove N. (2025). Reducing laboratory delays in blood culture pathogen identification: A quality improvement project. BMJ Open Qual..

[33] Liu W., Liao K., Wu J., Liu S., Zheng X., Wen W., Fu L., Fan X., Yang X., Hu X. (2024). Blood culture quality and turnaround time of clinical microbiology laboratories in Chinese Teaching Hospitals: A multicenter study. J. Clin. Lab. Anal..

[34] Royal College of Physicians of London (2012). National Early Warning Score (NEWS): Standardising the Assessment of Acute-Illness Severity in the NHS.

[35] Royal College of Physicians of London (2017). National Early Warning Score (NEWS) 2. https://www.rcp.ac.uk/improving-care/resources/national-early-warning-score-news-2/.

[36] Bekeris L.G., Tworek J.A., Walsh M.K., Valenstein P.N. (2005). Trends in blood culture contamination: A College of American Pathologists Q-Tracks study of 356 institutions. Arch. Pathol. Lab. Med..

